# Slow‐growing pedunculated nodule

**DOI:** 10.1002/ccr3.5485

**Published:** 2022-02-18

**Authors:** Chaima Kouki, Nadine Kammoun, Khadija Sellami, Emna Bahloul, Manel Mallouli, Lobna Ayadi, Tahya Boudaouara, Hamida Turki

**Affiliations:** ^1^ Department of Dermatology Hospital of Hedi Chaker Sfax Tunisia; ^2^ Department of Anatomopathology Hospital of Habib Bourguiba Sfax Tunisia

**Keywords:** giant basal cell carcinoma, pedenculated basal cell carcinoma

## Abstract

There have been limited reported cases of pedunculated basal cell carcinoma(BCC). Our case is original, and it combines two rare aspects of CBC: Pedunculated and giant. This uncommon aspect is rarely encountered.

## Graphical abstract

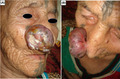

A 94‐year‐old patient with no medical history presented with a tumor of the nose that had progressed for 3 years. On examination, he was phototype II. There was an exophytic tumor, ulcerated in the center of size 7 cm and located on the right nostril wing (Figure 1). Surgical excision the lesion was performed. A pedunculated giant basal cell carcinoma (BCC) was diagnosed with the large basaloid tumor cell with peripheral palisading and retraction artifact and dilated vascular spaces on histopathology. (Figure 2).

**FIGURE 1:**
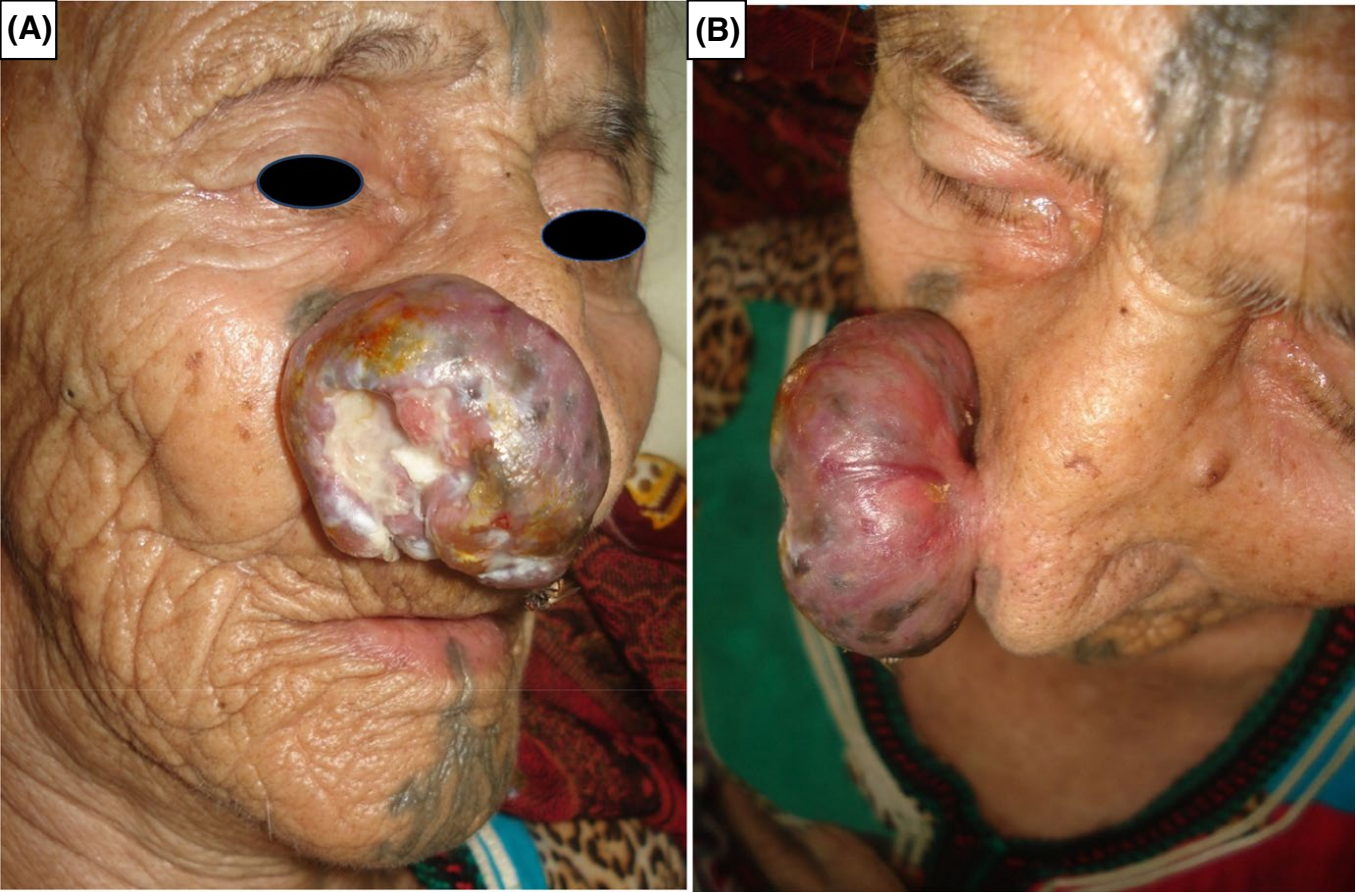
(A and B) Exophytic tumor, ulcerated in the center and pedenculated, 7 cm long in the axis of the right nasal ala

**FIGURE 2:**
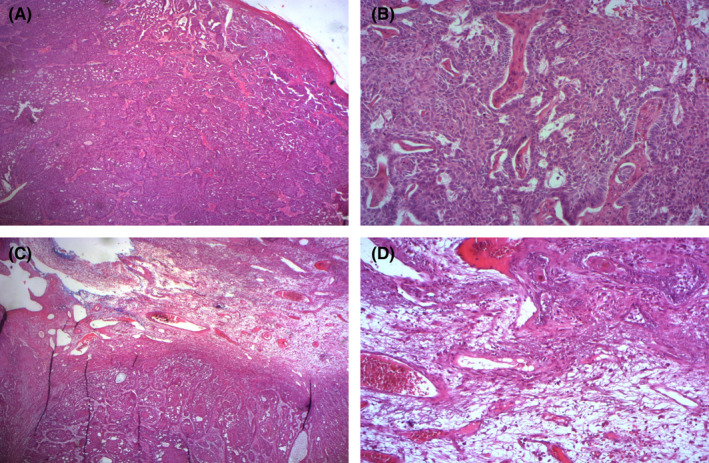
Pedunculated basal cell carcinoma: (A) Aspect of tumor proliferation of basaloid type (HE×25). (B) Basophilic tumor cells with palisade arrangement and retraction of the stroma (HE×100). (C) Infiltration of the pedicle by tumor casings (HE×50). (D) Significant vascularization of the tumor axis (HE×200)

Pedunculated basal cell carcinoma (BCC) is a rare entity.^1^ It is characterized by a polypoid appearance and a pedicle, which attaches it to the skin. Histologically, it is also distinguished by a pedunculated exophytic appearance and by restriction of tumor aggregations to the polypoid area.^1^ On the contrary, giant BCC (GBCC) accounts for 1% of all BCCs. GBCC is characterized by a size greater than 5 cm and an aggressive histological profile.^2^ It occurs most often in the cephalic extremity.

Our case is original, and it combines two rare aspects of CBC: Pedunculated and giant. This uncommon aspect is rarely encountered.

## CONFLICT OF INTEREST

None.

## AUTHOR CONTRIBUTIONS

Dr chaima kouki wrote the manuscript, is the guarantor of the content of the manuscript, and included the data and analysis. Dr kammoun nadine contributed to analysis and interpretation of data and revised it critically. Dr Sellami khadija contributed to interpretation of data and revision of the manuscript. Dr Bahloul emna contributed to data collection. Dr Mallouli manel and dr lobna ayadi provide the antomopathological figures. Dr Hamida Tuki and Dr boudawara tahya contributed to final approval of the version of the manuscript to be submitted.

## ETHICAL APPROVAL

Ethics statement was approved for this study.

## CONSENT

Written informed consent was approved by all authors. Written informed consent was also obtained from the patient to publish this report in accordance with the journal's patient consent policy.

## Data Availability

None.
